# A short perinuclear amphipathic α-helix in Apq12 promotes nuclear pore complex biogenesis

**DOI:** 10.1098/rsob.210250

**Published:** 2021-11-24

**Authors:** Wanlu Zhang, Azqa Khan, Jlenia Vitale, Annett Neuner, Kerstin Rink, Christian Lüchtenborg, Britta Brügger, Thomas H. Söllner, Elmar Schiebel

**Affiliations:** ^1^ Zentrum für Molekulare Biologie der Universität Heidelberg, DKFZ-ZMBH Allianz, Im Neuenheimer Feld 282, 69120 Heidelberg, Germany; ^2^ Biochemie-Zentrum der Universität Heidelberg, Im Neuenheimer Feld 328, 69120 Heidelberg, Germany

**Keywords:** APQ12, BRR6, nuclear pore complex, nuclear envelope, BRL1, nuclear pore complex biogenesis

## Abstract

The integral membrane protein Apq12 is an important nuclear envelope (NE)/endoplasmic reticulum (ER) modulator that cooperates with the nuclear pore complex (NPC) biogenesis factors Brl1 and Brr6. How Apq12 executes these functions is unknown. Here, we identified a short amphipathic α-helix (A*α*H) in Apq12 that links the two transmembrane domains in the perinuclear space and has liposome-binding properties. Cells expressing an *APQ12* (*apq12-ah*) version in which A*α*H is disrupted show NPC biogenesis and NE integrity defects, without impacting Apq12-ah topology or NE/ER localization. Overexpression of *APQ12* but not *apq12-ah* triggers striking over-proliferation of the outer nuclear membrane (ONM)/ER and promotes accumulation of phosphatidic acid (PA) at the NE. Apq12 and Apq12-ah both associate with NPC biogenesis intermediates and removal of A*α*H increases both Brl1 levels and the interaction between Brl1 and Brr6. We conclude that the short amphipathic α-helix of Apq12 regulates the function of Brl1 and Brr6 and promotes PA accumulation at the NE possibly during NPC biogenesis.

## Graphical abstract

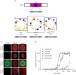

## 1. Introduction

The nuclear envelope (NE) is a double membrane consisting of the outer nuclear (ONM) and inner nuclear (INM) membranes that surround and protect the nucleus. The ONM is continuous with the endoplasmic reticulum (ER), contains attached ribosomes, carries attachment sites for cytoskeletal elements and shares components with the ER (reviewed in [1]). In addition, the ER and ONM are the sites of triacylglycerol (TAG) and steryl ester lipid biosynthesis [2]. These lipids are essential for membrane growth, and, in case of TAG, also for energy storage in the form of cytoplasmic lipid droplets [3,4]. The INM is involved in genome stability, chromatin organization and regulation of gene expression [5–7]. A recent publication indicated that TAGs are also synthesized at the INM before being incorporated into nuclear lipid droplets [8]. Consistent with these distinct functions, the INM and ONM are specified by proteins and lipids that vary from one another.

The NPC is a large oligomeric complex containing about 30 different proteins that is embedded at sites at which the INM and ONM fuse. The NPCs facilitate the transport of proteins and ribonucleoproteins between the cytoplasm and the nucleoplasm [6]. In human cells and other eukaryotes undergoing an open mitosis, NPCs assemble via two mechanistically distinct pathways [9]. The post-mitotic pathway promotes the assembly of NPCs on the decondensing chromatin, shortly after anaphase onset [10]. During interphase, NPCs assemble by a distinct inside-out mechanism starting from within the nucleus at the INM, the so-called interphase pathway [11–13]. In budding yeast, with its closed mitosis, the interphase pathway is the only mechanism to assemble NPCs.

NPC biogenesis intermediates of the interphase pathway have been described in human cells by electron tomography [11]. In wild-type (WT) yeast cells, NPC intermediates have not been observed, probably because NPC assembly is relatively fast and infrequent (approx. 2 assembly events per minute per cell [13]). However, mutations in genes coding for several nucleoporins (Nups) lead to the accumulation of so-called herniations: deformations of the INM that are probably filled with Nups [14–17]. Recently, we suggested that at least some of these herniations arise from defective steps of NPC biogenesis; for example, the failure of the fusion of the INM with the ONM during the assembly process [5,12].

Mechanistic principles of NPC biogenesis by the interphase pathway are poorly understood. In mammalian cells, early factors involved in interphase NPC biogenesis include Y-complex subunits NUP107 and NUP133, the INM protein SUN1 and the transmembrane nucleoporin POM121 [9,18–20]. In yeast, the two paralogous integral NE proteins Brl1 and Brr6 function in NPC biogenesis [12,21–25]. Loss of function of Brl1 (Brr6 like protein No. 1) or Brr6 (bad response to refrigeration) gives rise to herniations without impacting the insertion or function of pre-existing NPCs indicating that both proteins are specifically required for NPC assembly but not for the maintenance of assembled NPCs. Consistent with this notion, Brl1 and Brr6 only associate with NPC intermediates and not with fully assembled NPCs [12]. Brl1 and Brr6 physically and genetically interact with the small integral NE/ER protein Apq12 (apical growth revealed by quantitative morphological analysis) to reveal synergistic regulation by all three of these proteins [21,23,26]. Cells with a deletion in *APQ12* (*apq12Δ*) are cold sensitive for growth. Puzzlingly, at 37°C *apq12Δ* cells show a defect in NPC biogenesis despite relatively normal growth [27].

A clear functional link between *BRL1* and NPC assembly has been revealed by genetic suppression of the physical interaction between the FG repeat containing Nup116 and the inner ring protein Nup188 that has a scaffolding function during NPC biogenesis leading to the accumulation of herniations in *NUP116* and *NUP188* defective cells [5,28]. Interestingly, the INM/ONM fusion defect of these scaffolding defective yeast cells was efficiently suppressed by overexpression of *BRL1* but not of *BRR6* [12]. This suggests a role of Brl1 in INM/ONM fusion and also shows that Brl1 and Brr6 have distinct functions during NPC biogenesis.

Here, we report that Apq12 carries a short perinuclear amphipathic alpha-helix (A*α*H) connecting the two transmembrane domains. The A*α*H peptide binds to liposomes and amino acid substitutions that disrupt the amphipathic nature of the peptide abolish the binding to liposomes. Cells carrying an Apq12 with a defective A*α*H (*apq12-ah*) are cold sensitive for growth, show NPC biogenesis defects and disrupted NE even though the distribution of the mutant protein is unaffected and it assumes the correct topology within the membrane. Overexpression of *APQ12* triggers strong over-proliferation of the ONM and the accumulation of phosphatidic acid (PA) at the NE in a manner that is reliant upon the presence of a functional A*α*H. Apq12 associates with NPC biogenesis intermediates at bent INM segments, a localization that does not require a functional A*α*H. In addition, the *apq12-ah* mutant shows elevated Brl1 and Brr6 levels and enhanced interaction between Brl1 and Brr6. Taken together, these data place the A*α*H of Apq12 into a strategic position to coordinate PA accumulation at the NE, Brl1–Brr6 interaction and NPC biogenesis.

## 2. Results

### 2.1. Apq12 carries a short lipid-binding AαH between the two transmembrane domains

**Figure 1.**
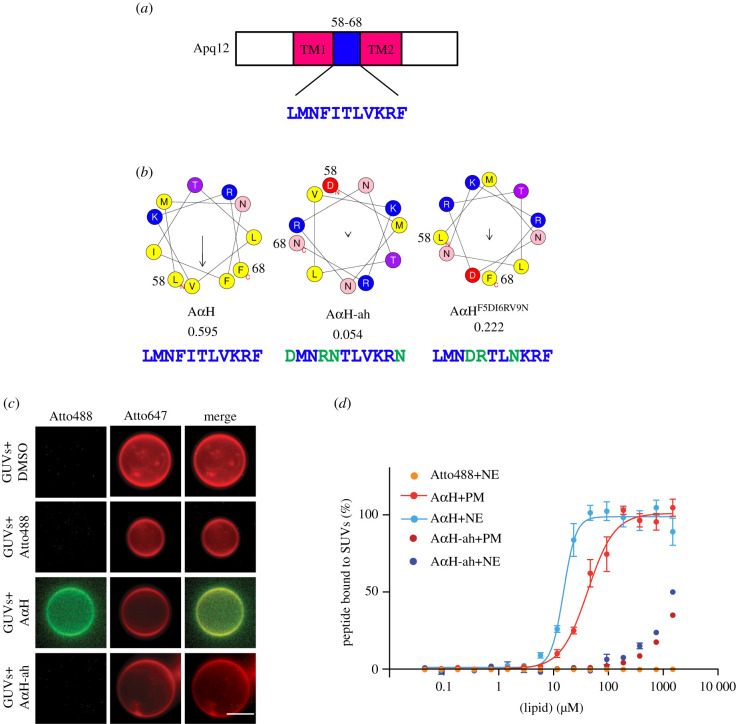
Apq12 contains an amphipathic helix in the luminal domain. (*a*) Domain organization of Apq12. The first transmembrane (TM1) domain, an A*α*H (blue) and the second transmembrane (TM2) domain are indicated. (*b*) Heliquest predictions of the A*α*H, A*α*H-ah and A*α*H^F5DI6RV9^^N^ helices along with their hydrophobic moments. Heliquest calculates the physicochemical properties of an α-helix [29]. The amino acids marked in green indicate amino acid changes introduced in A*α*H in order to reduce the hydrophobic moment (value given below). The amino acid number corresponding to Apq12 indicates start and end of the α-helix. The arrow inside the helix indicates the hydrophobic moment. (*c*) Binding of Atto488 labelled synthetic A*α*H and A*α*H-ah peptides to Atto647 labelled GUVs, *in vitro*. DMSO and Atto488 dye are used as controls. Scale bar: 5 µm. (*d*) Liposomes with different lipid compositions were titrated against Atto488 labelled A*α*H and A*α*H-ah peptides and the Atto488 dye as control. The data were normalized to the amount of bound peptide and the *K_D_* value of A*α*H peptide-liposome interaction was calculated from the Hill equation (PM: *K_D_* = 58.90 ± 5.35 µM, NE: *K_D_* = 15.90 ± 0.91 µM). The A*α*H-ah peptide and the Atto488 dye (together with NE lipids; same result was obtained with PM lipids) show no or only very weak MST signals. Values are given as means ± s.e.m, *n* = 3.

Recently, we have shown that the integral membrane protein Brl1 associates with NPC assembly intermediates and may promote fusion of the ONM with the INM during NPC biogenesis [12]. Brl1 interacts and cooperates with the integral membrane protein Apq12 [21]. In order to gain an understanding of the molecular roles of Apq12 (figure 1*a*), we sought functional elements within this protein. The AmphipaSeeK program predicted an amphipathic α-helix (A*α*H) with two positively charged amino acids (Lys and Arg) on the hydrophilic side and hydrophobic amino acid residues on the opposite side of the helix (figure 1*b*; ‘A*α*H’) [30], between the two TM domains of Apq12 (figure 1*a*).

The positively charged amino acids of an A*α*H (figure 1*b*, marked blue) have the ability to interact with polar–apolar lipid surfaces while the hydrophobic interface (figure 1*b*, yellow) may interact with the aliphatic chains of the lipids [31]. Consistent with this lipid binding prediction, we found that the Atto488 labelled synthetic Apq12 peptide (A*α*H) bound to giant unilamellar vesicles (GUVs; 1–10 µm in diameter; figure 1*c*). As control for the binding, we used the Atto488 dye alone, and introduced amino acid changes in the Atto488-labeled Apq12 peptide (A*α*H-ah) that abolished the helical hydrophobic moment, as an indication of the amphiphilicity of the helix [32], from 0.595 to 0.054 (figure 1*b*). The Atto488 dye and the A*α*H-ah peptide both failed to bind to liposomes (figure 1*c*).

To analyse the binding efficiency of A*α*H peptide in a more quantitative manner, SUV (small uni-lamellar vesicles; 80–120 nm; electronic supplementary material, figure S1A) bound peptide was separated from the unbound peptide through a nycodenz gradient. Using SUVs composed of NE and plasma membrane (PM) lipids, we tested the impact of the lipid composition. NE-derived SUVs showed a higher A*α*H peptide binding efficiency compared to the PM SUVs (electronic supplementary material, figure S1B). The Atto488 dye and the A*α*H-ah peptide did not bind to the SUVs in this experimental regime (electronic supplementary material, figure S1B). In addition, the peptide without liposomes was unable to float through the nycodenz gradient (electronic supplementary material, figure S1B, control).

Next, SUV binding of the A*α*H peptide was quantified using microscale thermophoresis (MST) measurements. The *K_D_* of A*α*H peptide binding to NE-lipid derived SUVs was 16 µM (figure 1*d*). The binding affinity of the A*α*H peptide to PM derived SUVs was decreased to *K_D_* = 59 µM (figure 1*d*). Importantly, A*α*H-ah peptide failed to bind to any of these SUVs with a measurable *K_D_*. Thus, the A*α*H peptide binds to liposomes depending on its amphipathic nature and the lipid composition of the liposomes. This result indicates that the A*α*H of Apq12 directly interacts with membranes.

### 2.2. The A*α*H of Apq12 localizes in the perinuclear space and is not required for subcellular localization and topology of Apq12

**Figure 2.**
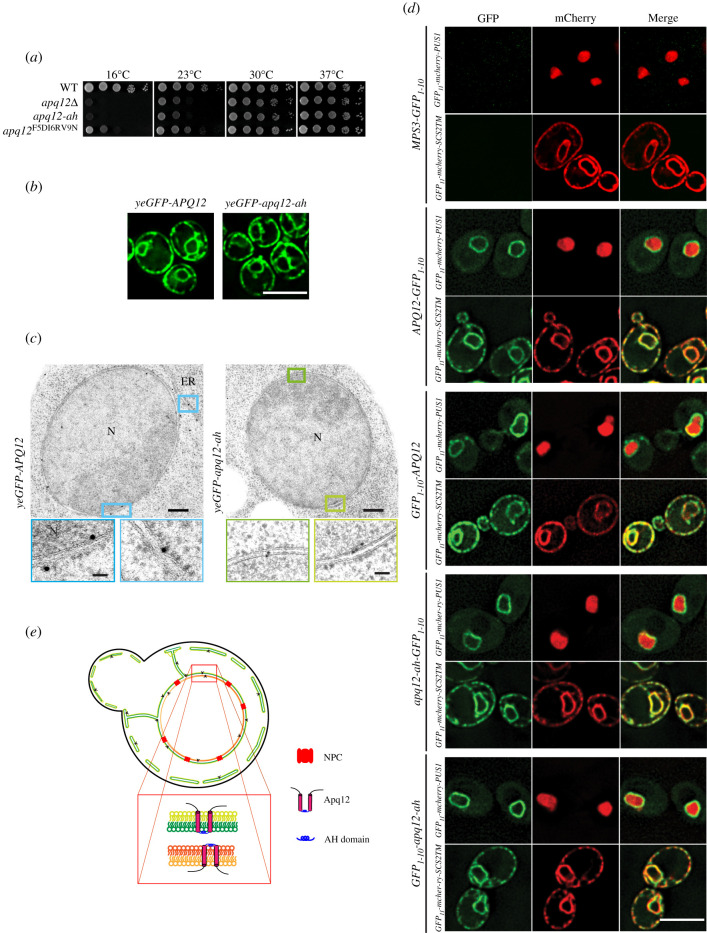
The A*α*H of Apq12 is located in the perinuclear space and does not influence the subcellular localization and topology. (*a*) Growth test of WT *APQ12*, *apq12Δ*, *apq12-ah* and *apq12^F5DI6RV9^^N^* mutants at the indicated temperatures. 10-fold serial dilutions were spotted onto YPAD plates. (*b*) Localization of yeGFP-Apq12 and yeGFP-apq12-ah were analysed by fluorescence microscopy. Scale bar: 5 µm. (*c*) yeGFP-Apq12 and yeGFP-apq12-ah localization by immuno-EM. Gold particles (10 nm) indicate the localization of yeGFP-Apq12 and yeGFP-apq12-ah at the NE and ER. The rectangles indicate the enlargements that are shown underneath. ER, endoplasmic reticulum; N, nucleus. Scale bars: 200 nm and enlargements 50 nm. (*d*) Strains carrying C- and N-terminal fusions of Apq12 and Apq12-ah with GFP_1-10_ were imaged to check for reconstitution of GFP with GFP_11_ from GFP_11_-mCherry-Scs2TM (ER reporter; GFP_11_ in the cytoplasm) and GFP_11_-mCherry-PUS1 (nuclear reporter). Mps3-GFP_1-10_ is used as a negative control. Scale bar: 5 µm. (*e*) Localization and topology model for Apq12.

We analysed whether the amphipathic nature of the A*α*H is important for the function of Apq12. Since *apq12Δ* mutants show a growth defect at 16°C [26], we tested *APQ12*, *apq12Δ, apq12-ah* and an additional A*α*H *APQ12* mutant*, apq12^F5DI6RV9N^*, for growth at different temperatures. The helical hydrophobic moment of the A*α*H was decreased in A*α*H^F5DI6RV9N^ to 0.222 and therefore has an intermediate value between the WT A*α*H of Apq12 and A*α*H-ah of Apq12-ah (figure 1*b*). *apq12-ah* mutant completely failed to grow at 16°C and showed reduced growth at 23°C, similar to the *apq12Δ* cells (figure 2*a*). By contrast, growth of *apq12^F5DI6RV9N^* mutant cells was only reduced, but not completely abolished, at 16°C (figure 2*a*). Therefore, all further experiments were performed with *apq12-ah* cells. Thus, the amphipathic nature of the A*α*H in Apq12 is important for cell growth at lower temperatures.

We next analysed whether the subcellular localization of Apq12 requires the A*α*H. Consistent with published data on Apq12 distribution [21], yeGFP-Apq12 localized along the NE and the cell periphery, and a location that is probably the peripheral ER (figure 2*b*). Based on the resolution provided by the fluorescence microscope, similar localizations were observed for yeGFP-Apq12-ah (figure 2*b*). Thus, the integrity of the A*α*H of Apq12 is not important for the subcellular distribution of the protein.

Immuno-electron microscopy of yeGFP-Apq12 and yeGFP-Apq12-ah using GFP antibodies and protein A-gold detected both proteins along the INM, ONM, cytoplasmic and cortical ER (figure 2*c*; electronic supplementary material, figure S1C). Apq12 was detected with the same frequency at the INM as at the ONM (electronic supplementary material, figure S1C). For Apq12-ah, there was a mild enrichment at the INM over the ONM (electronic supplementary material, figure S1C). In addition, yeGFP-Apq12 and yeGFP-Apq12-ah associated with 10% and 15% of NPCs, respectively, while most NPCs were not labelled (electronic supplementary material, figure S1D). This may indicate a transient association of the proteins with assembling NPCs, as is the case for Brl1 and Brr6 [12]. In summary, Apq12 and Apq12-ah show similar subcellular localizations.

Apq12 is a protein of the NE and the ER with two predicted membrane spanning regions (TM1 and TM2) that are connected by the A*α*H (figure 1*a*). To determine the topology of Apq12 and whether it requires a functional A*α*H, we measured the accessibility of the N- and C-terminus of Apq12 and Apq12-ah by two approaches. First, we used the split GFP system for assessment of the localization of the N- and C-termini of Apq12 [33]. The overlapping localization of GFP_11_ and GFP_1-10_ restores GFP fluorescence. Thus, by combining GFP_1-10_ tagged versions of a protein with nuclear and cytoplasmic GFP_11_ localized proteins, we can determine the topology of a protein. As a control, we used the perinuclear space localization of the C-terminus of the SUN-domain protein Mps3-GFP_1-10_ that failed to restore a fluorescent GFP signal when co-expressed with the ER and ONM localized GFP_11_-mCherry-Scs2TM that carries GFP_11_ at the ONM/ER on the cytoplasmic side and the nuclear GFP_11_-mCherry Pus1 (figure 2*d*, top panel) consistent with published data [33]. Interestingly, Apq12-GFP_1-10_ and GFP_1-10_-Apq12 combined with GFP_11_-mCherry Pus1 resulted in a green fluorescent NE signal and, in the case of GFP_11_-mCherry-Scs2TM, a NE/ER signal. These data show that the N- and C-termini of Apq12 are localized to either the cytoplasm or the nucleoplasm depending on whether Apq12 is at the ONM/ER or INM, respectively (figure 2*d*, middle). Very similar results were obtained for Apq12-ah (figure 2*d*, bottom).

As a further probe of Apq12 topology, we took advantage of the fact that biotin ligases are not contained in the perinuclear space, and assessed the ability of the N- or the C-termini of Apq12 and Apq12-ah to be biotinylated when fused with the histidine-biotin-histidine (HBH) tag as a topology marker [12]. Pom152 that contains a short cytosolic N-terminal region, one TM domain and a C-terminal region in the perinuclear space was used as a control [34]. Consistent with the topology of Pom152, only the N-terminal HBH tag but not the C-terminal tag of Pom152 was biotinylated, demonstrating that the HBH biotinylation approach identifies the topology of NE proteins correctly (electronic supplementary material, figure S1E). Both the N- and C-termini of Apq12 and Apq12-ah were biotinylated when fused to the HBH tag that carries a biotin acceptor site (electronic supplementary material, figure S1E), confirming the findings from the split GFP approach.

In conclusion, since N- and C-termini of Apq12 localized to either the cytoplasm or nucleoplasm, the A*α*H resides in the perinuclear space where it connects the two TM domains (figure 2*e*). A functional A*α*H is not required for this topological arrangement of Apq12.

### 2.3. The A*α*H of Apq12 is important for NE integrity, NPC biogenesis and lipid homeostasis

**Figure 3.**
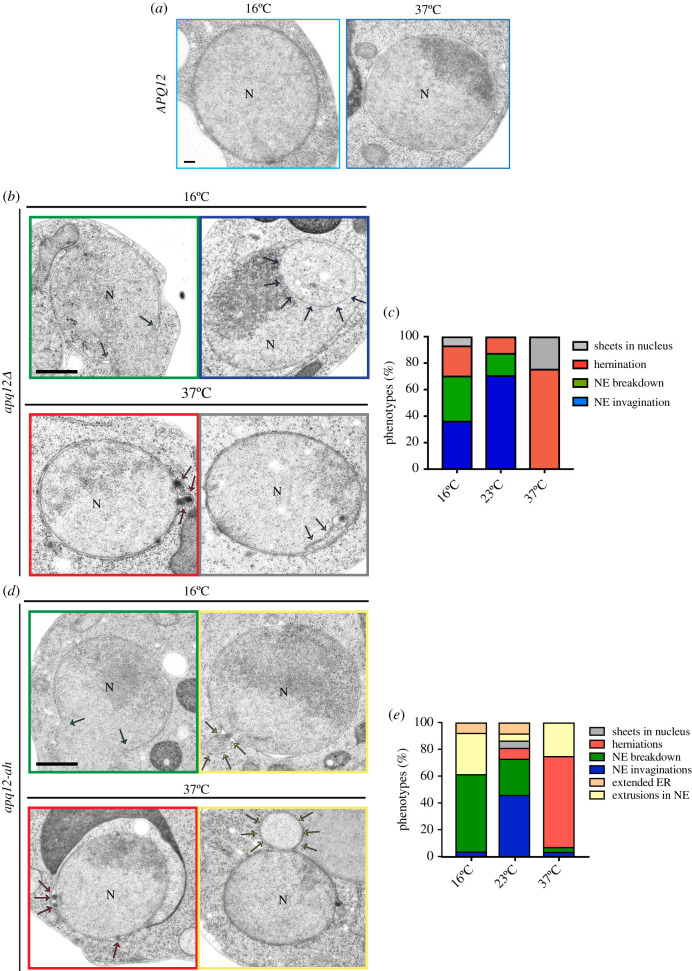
Phenotypes of *apq12Δ* and *apq12-ah* mutants. (*a*) EM analysis of *APQ12* WT cells incubated at the indicated temperatures. (*b*) EM analysis of *apq12Δ* mutants incubated at the indicated temperatures. The arrows point towards the phenotype that is indicated by the colour code encircling the picture. (*c*) Quantification of phenotypes from (*b*). Cells (*n* = 44/16°C, 40/23°C and 57/37°C) were analysed per temperature. (*d*) EM analysis of *apq12-ah* mutants grown at the indicated temperature. The arrows point towards the phenotype that is indicated by the colour code encircling the picture. (*e*) Quantification of phenotypes from (*d*). Cells (*n* = 27/16°C, 39/23°C and 28/37°C) were analysed per temperature. (*a*, *b* and *d*) N, nucleus. Scale bars: (*a*) 100 nm; (*b*,*d*) 500 nm.

In order to understand how loss or impairment of the Apq12 function affects the NE and NPCs, we analysed the phenotypes of *APQ12*, *apq12Δ* and *apq12-ah* cells by electron microscopy (EM). As expected, *APQ12* wild-type cells had spherical nuclei with intact NE (figure 3*a*). Herniations as an indication of a defect in NPC biogenesis were detected as a major phenotype in *apq12Δ* (figure 3*b,c*, nucleus marked by red square) and *apq12-ah* mutants at 37°C (figure 3*d,e*, nucleus marked by red square). About 70% of the *apq12Δ* mutants incubated at 23°C showed invaginations of the NE (figure 3*c*). Herniations were relatively infrequent at this growth temperature. Invaginations of the NE and NE breakdown were the major defects of the *apq12-ah* mutant at 23°C (figure 3*e*). At 16°C, the major defects of *apq12Δ* cells were NE invaginations and NE breakdown followed by herniations (figure 3*b,c*). *apq12-ah* mutant showed NE breakdown and extrusions as major defects at 16°C (figure 3*d,e*). Taken together, *apq12Δ* and *apq12-ah* mutants show phenotypic variations indicating that inactivation of the A*α*H does not cause the complete loss of Apq12 function. In addition, the consequence of Apq12 or A*α*H loss depends on the temperature. The rupture of the NE explains the lethality of *apq12Δ* and *apq12-ah* cells at 16°C. Loss of A*α*H function triggers a defect in NPC biogenesis at 37°C.

It has been suggested that Apq12 has a function in lipid homeostasis [21], which might then account for the NE and NPC defects of *apq12* and *apq12-ah* mutants. Therefore, we directly asked whether the A*α*H of Apq12 has an impact on lipid homeostasis, by incubating WT, *apq12Δ* and *apq12-ah* cells at 16°C, 30°C and 37°C, and assessing their cellular lipid content by mass spectrometry. Overall *apq12Δ* and *apq12-ah* mutants showed comparable lipid changes relative to WT (electronic supplementary material, figure S2). The increase in ergosteryl ester (EE) and TAG, and a decrease in ergosterol (Erg) and most species of glycerophospholipids (GPL), were prominent phenotypes in *apq12Δ* and *apq12-ah* mutants (electronic supplementary material, figure S2A and B), indicating a lipid metabolism flow from membrane lipids to storage lipids. In addition, we observed a significant decrease of double bonds in GPL in *apq12Δ* and *apq12-ah* mutants (electronic supplementary material, figure S2C). Moreover, the chain length (greater than 34) in GPL was significantly increased in both *APQ12* mutants (electronic supplementary material, figure S2D). The reduction of membrane lipids, the decrease in the number of double bonds and the increase of chain length in GPL indicate a decrease in membrane fluidity in *apq12Δ* and *apq12-ah* mutants, explaining the defects in NE breakdown at 16°C when the flexibility of membrane lipids will be reduced by the reduction in kinetic energy. These data suggest that the A*α*H of Apq12 does play a role in maintaining lipid homeostasis.

### 2.4. Increased Apq12 levels are toxic to cells and cause the mis-localization of the NPC biogenesis factors Brl1 and Brr6 and the ER proteins Sec63 and Ole1

**Figure 4.**
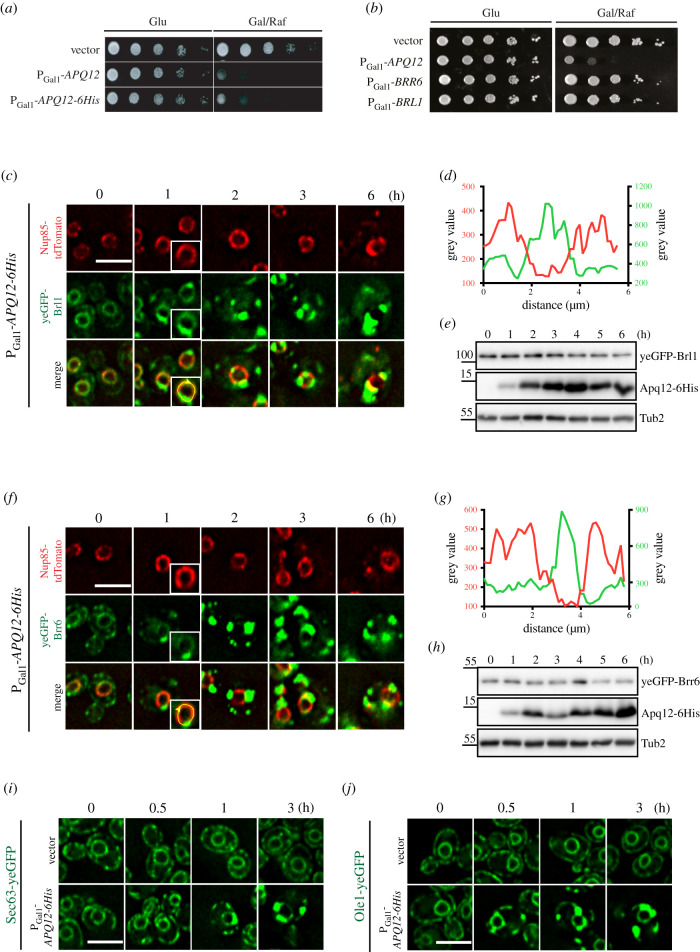
Overexpression of *APQ12* causes the mislocalization of ER proteins. (*a*) Overexpression of *APQ12* is toxic for the cells. WT cells with the vector control, P_Gal1_-*APQ12* or P_Gal1_-*APQ12-6His* were spotted in 10-fold serial dilutions onto YPAD (Glu) and YARaf/Gal (Gal/Raf) plates at 30°C. (*b*) Of the *APQ12*, *BRL1*, *BRR6* module only *APQ12* overexpression is toxic. WT cells with the vector control, P_Gal1_-*APQ12*, P_Gal1_-*BRR6* and P_Gal1_-*BRL1* were spotted in 10-fold serial dilutions onto YPAD (Glu) and YARaf/Gal (Gal/Raf) plates at 30°C. (*c*) Overexpression of *APQ12* causes mislocalization of yeGFP-Brl1. Cells with either the vector control (electronic supplementary material, figure S3C) or P_Gal1_-*APQ12-6His* were incubated with galactose for the indicated times. The boxed cell at 1 h is a two-fold enlargement of the selected cell. (*d*) Line scan along the NE of a P_Gal1_-*APQ12-6His yeGFP-BRL1 NUP85-tdTomato* cell (enlarged boxed cell in (*c*)) incubated for 1 h with galactose. It shows that yeGFP-Brl1 and Nup85-tdTomato are localized on separate domains along the NE. (*e*) Immunoblot of P_Gal1_-*APQ12-6His yeGFP-BRL1 NUP85-tdTomato* cells. The pGal1 promoter was induced by the addition of galactose (*t* = 0). Samples were taken after the indicated times. Tub2 is a loading control. Apq12-6His was detected by anti-His antibodies. (*f*) Overexpression of *APQ12* causes mislocalization of yeGFP-Brr6 and Nup85-tdTomato. Cells with either the vector control (electronic supplementary material, figure S3D) or P_Gal1_-*APQ12-6His* were incubated with galactose for the indicated times. The boxed cell at 1 h is a twofold enlargement of the selected cell. (*g*) Line scan along the NE of a P_Gal1_-*APQ12-6His yeGFP-BRR6 NUP85-tdTomato* cell (enlarged cell in (*f*)) incubated for 1 h with galactose. (*h*) Immunoblot of P_Gal1_-*APQ12-6His yeGFP-BRR6 NUP85-tdTomato* cells. The pGal1 promoter was induced by the addition of galactose (*t* = 0). Samples were taken after the indicated times. Tub2 is a loading control. (*i,j*) Overexpression of *APQ12* causes mislocalization of the ER protein Sec63-yeGFP (*i*) and Ole1-yeGFP (*j*). Cells with either the vector control or P_Gal1_-*APQ12-6His* were incubated with galactose for the indicated time. (*c,f,i* and *j*) Scale bars: 5 µm.

To gain deeper insights into the function of Apq12, we overexpressed *APQ12* using the galactose-inducible P_Gal1_ promoter and followed the growth of these modified yeast cells. Because we lack an Apq12 antibody, we tagged Apq12 with 6His (Apq12–6His) to support immuno-detection of the fusion protein. Overexpression of *APQ12* and *APQ12-6His* was equally toxic for cells (figure 4*a*). Such overexpression toxicity was not observed for the partner proteins *BRL1* and *BRR6*, which also encode integral membrane proteins (figure 4*b*). Thus, of the three proteins in this functional module, only *APQ12* has toxic consequences upon overproduction.

We next tested whether overexpression of *APQ12* and *APQ12-6His* impact on NE structure and function. We started by analysing the impact of *APQ12* over-expression on the distribution of the NPC biogenesis factors Brl1 and Brr6 (figure 4*c–h*; electronic supplementary material, figure S3A and B). In vector control cells, yeGFP-Brl1 and yeGFP-Brr6 exhibited a uniform distribution throughout the NE (electronic supplementary material, figure S3C and D). By contrast, 1 h of induction of the P_Gal1_ promoter to elevate Apq12 levels led to dense clustering of Brl1 and Brr6 on the NE (figure 4*c–h*; electronic supplementary material, figure S3A and B). These clusters were devoid of the NPC marker Nup85-tdTomato (figure 4*c,d,f,g*; electronic supplementary material, figure S3A and B). Clustering of yeGFP-Brl1 and yeGFP-Brr6 in response to *APQ12-6His* overexpression was also seen in time-lapse experiments (electronic supplementary material, figure S3E and F). Because P_Gal1_-*APQ12* and P_Gal1_-*APQ12-6His* caused similar defects, we used P_Gal1_-*APQ12-6His* in further experiments.

We next asked how *APQ12-6His* overexpression affected the distribution of proteins at the NE/ER and INM, using the NE/ER proteins Sec63 and Ole1, the INM protein Asi3 and the ER luminal marker dsRED-HDEL as exemplars to test the impact upon NE/ER proteins [35,36]. As seen for yeGFP-Brl1 and yeGFP-Brr6, the smooth distribution of Sec63-yeGFP and Ole1-yeGFP along the NE was transformed into clusters as early as 1 h of P_Gal1_-*APQ12-6His* induction (figure 4*c,f,i,j*). The localization of INM protein Asi3-yeGFP was initially unaffected by *APQ12* overexpression, at the 1 h time point, however, by 3 h of P_Gal1_ induction, modest clustering of Asi3-yeGFP along the NE emerged (electronic supplementary material, figure S4A; white arrows). The ER marker dsRED-HDEL, that in vector control cells uniformly stained the NE and the cortical ER, started to accumulate in clusters within one hour of P_Gal1_-*APQ12-6His* induction (electronic supplementary material, figure S4B). Thus, increased Apq12 levels appear to impact upon the subcellular localization of ONM/ER proteins more strongly than the INM protein Asi3.

### 2.5. The luminal A*α*H of Apq12 contributes to toxicity

**Figure 5.**
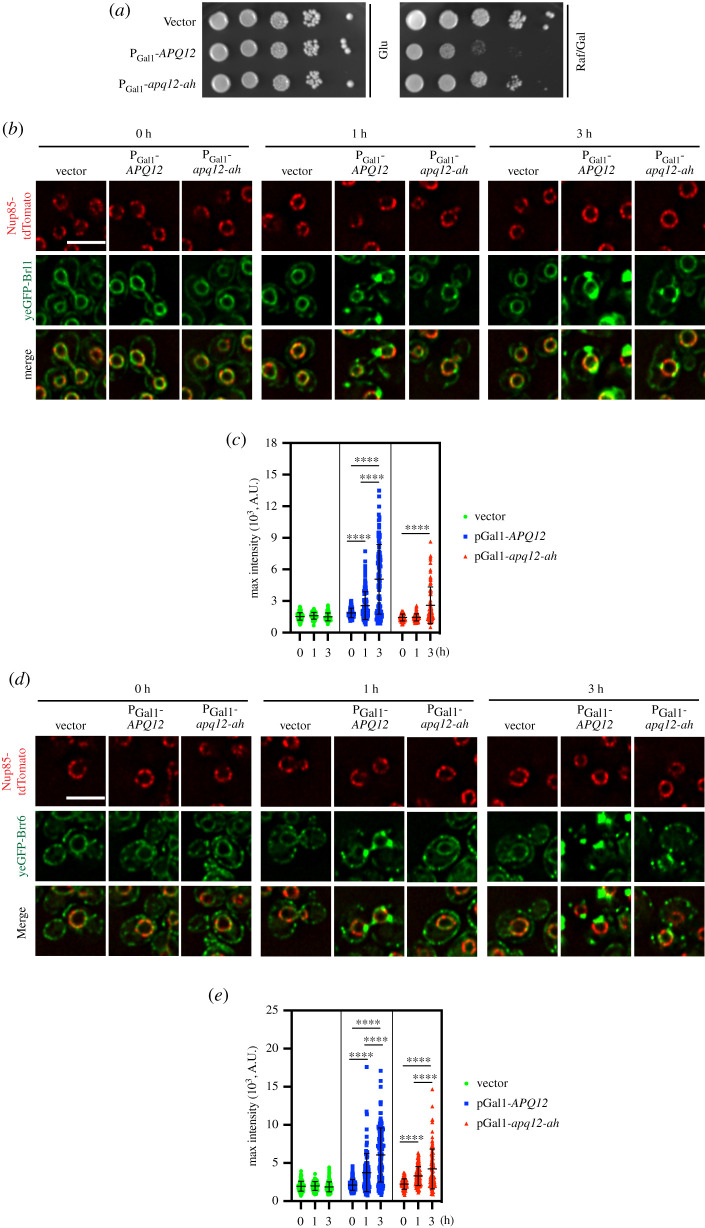
The A*α*H of Apq12 is important for membrane remodelling. (*a*) Inactivation of the A*α*H of Apq12 reduces overexpression toxicity. Growth test of cells containing P_Gal1_-*apq12-ah*, in comparison to P_Gal1_-*APQ12* and vector control cells. Ten-fold serial dilutions of cells were spotted onto glucose (Glu) and galactose/raffinose (Gal/Raf) plates at 30°C. (*b–e*) Impact of *APQ12* and *apq12-ah* overexpression on (*b,c*) *yeGFP-BRL1 NUP85-tdTomato* and (*d,e*) *yeGFP-BRR6 NUP85-tdTomato* cells. (*c,e*) Quantifications of the maximum GFP intensity of individual cells from (*b*) and (*d*) over time. Two independent experiments were performed, and more than 60 cells were analysed per experiment for each time point. Values are given as means ± s.d. *t*-test ****p < 0.0001. (*b,d*) Scale bars: 5 µm.

We asked whether A*α*H is important for the deformation of the NE*.* P_Gal1_-*apq12-ah-6His* overexpression was less toxic than P_Gal1_-*APQ12-6His* (figure 5*a*) even though both proteins were overexpressed to similar levels (electronic supplementary material, figure S5A). Consistent with the strongly reduced toxicity of P_Gal1_-*apq12-ah-6His*, yeGFP-Brl1 and yeGFP-Brr6 clusters formed later and were notably less prominent in P_Gal1_-*apq12-ah-6His* cells compared to P_Gal1_-*APQ12-6His* cells (figure 5*b–e*). Thus, an intact A*α*H is a major factor in the toxic impact of *APQ12* overexpression.

### 2.6. Apq12 needs a functional A*α*H for the over-proliferation of the ONM and ER

**Figure 6.**
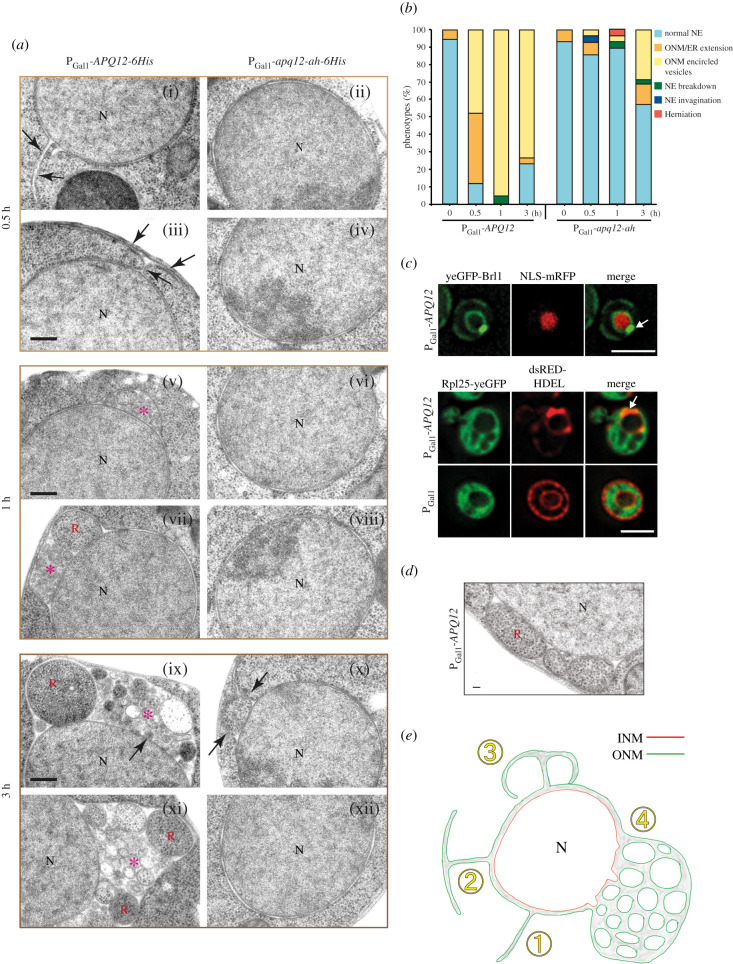
Overexpression of *APQ12* triggers hyper-proliferation of the ONM and ER. (*a*) EM analysis of WT cells expressing P_Gal1_-*APQ12-6His* (left) or P_Gal1_-*apq12-ah-6His* (right) by the addition of galactose for 0.5 h, 1 h and 3 h. Arrows are explained in the result section; red asterisks indicate ONMs encircled vesicles containing granular material. N, nucleus; R, ribosome-like particles. Scale bar: 200 nm. (*b*) Quantification of phenotypes from (*a*). *n* = 25 cells were analysed for each time point and strain. (*c*) P_Gal1_-*APQ12* was overexpressed for 3 h by the addition of galactose. Top panel: cells expressing yeGFP-Brl1 along with a nuclear marker NLS-mRFP. Bottom panel: cells expressing yeGFP tagged ribosomal subunit Rpl25 or containing the P_Gal1_ control, and an ER luminal marker dsRED-HDEL. Arrows indicate strong yeGFP-Brl1 and dsRED-HDEL signals at the NE. Scale bar: 3 µm. (*d*) Electron micrograph showing ribosome-like particles trapped in vesicles formed upon overexpression of *APQ12*. Scale bar: 50 nm. (*e*) Model for the formation of membrane structures in response to *APQ12* overexpression. See Results for details.

To understand how *APQ12* overexpression leads to the changes on the ONM and ER, we performed EM analysis of P_Gal1_-*APQ12-6His* and P_Gal1_-*apq12-ah-6His* cells. Before the addition of galactose, the NE had uniform spherical morphology (electronic supplementary material, figure S5B). Induction of P_Gal1_-*APQ12-6His* led to a shift in the number of ONM/ER extensions (figure 6*a*(i), arrow) that were, in some cases, connected to the cortical ER (figure 6*a*(ii)) from 5% to 40% (figure 6*b*). After 1 and 3 h of galactose addition, P_Gal1_-*APQ12-6His* cells contained ONM encircled vesicles containing granular material (figure 6*a*(v–xii), red asterisks; and figure 6*b*). We noticed a slight reduction of the severity of these phenotypes after 3 h of P_Gal1_-*APQ12-6His* overexpression. This may be related to adaptation or overexpression inactivation in some cells. By contrast, abnormal morphologies were not detected at the INM until 3 h after the induction of *APQ12* overexpression, whereupon the INM formed small buds into the luminal space of the ONM vesicles (figure 6*a*(ix), arrow). The consequences of *apq12-ah-6His* overexpression were less severe in comparison to *APQ12-6His*. P_Gal1_-*apq12-ah-6His* induction for 0.5 h and 1 h had no visible impact on the ONM or the ER in most cells (figure 6*a*(ii–viii)). Only 3 h of P_Gal1_-*apq12-ah-6His* induction caused proliferation of the ONM in approximately 25% of the cell sections (figure 6*a*(viii), arrows and figure 6*b*). This delayed accumulation of phenotypes in *apq12-ah-6His* cells is consistent with the reduced clustering of the *BRL1-yeGFP* and *BRR6-yeGFP* and impact on growth compared to *APQ12-6His* (figure 5).

Overexpression of *APQ12* causes the accumulation of large, granular ONM encircled vesicles. To investigate the origin of the content in these vesicles, we expressed P_Gal1_-*APQ12-6His* in cells with yeGFP tagged Brl1 as marker for the vesicles, and NLS-mRFP (mRFP fused to a nuclear localization sequence (NLS)) as nuclear marker. Overexpression of Apq12-6His induced yeGFP-Brl1 areas that were devoid of NLS-mRFP (figure 6*c*, top, marked by arrow) indicating that these vesicles did not contain nuclear components. In a complementing experiment, we expressed P_Gal1_-*APQ12-6His* in *RPL25-yeGFP dsRed-HDEL* cells with the green fluorescent ribosomal subunit as cytoplasmic marker and dsRed-HDEL as ONM/ER marker (figure 6*c*, bottom). Indeed, the dsRed-HDEL ONM extrusions co-localized with Rpl25-yeGFP (figure 6*c*, bottom; arrow), demonstrating that these vesicles contained cytoplasmic components including ribosomes. This was further suggested by EM analysis of P_Gal1_-*APQ12-6His* cells showing ONM encircled vesicles containing ribosome-like particles (figure 6*d*).

In a time-lapse experiment, we analysed the cellular distribution of Apq12-yeGFP and Apq12-ah-yeGFP upon P_Gal1_ expression (electronic supplementary material, figure S5C). Apq12-yeGFP accumulated most prominently at the NE extensions (electronic supplementary material, figure S5C, 30 min; arrowheads). After 40 min of induction Apq12-yeGFP localized in larger spots (electronic supplementary material, figure S5C; arrowheads), consistent with the accumulation of filled ONM deformations seen by EM (figure 6*a*). For the first 40 min of P_Gal1_ induction Apq12-ah-yeGFP showed comparable localizations to Apq12-yeGFP (electronic supplementary material, figure S5C). However, due to its defective A*α*H, Apq12-ah-yeGFP did not accumulate into larger spots. Analysis of P_Gal1_-*APQ12-yeGFP* after 0.5 and 3 h of galactose addition by immuno-EM detected Apq12-yeGFP locating at NE extensions and ONM enriched vesicles (electronic supplementary material, figure S5D). Thus, the enrichment of Apq12 at ONM/ER sites induces membrane proliferation.

When taken together, *APQ12* overexpression promotes extension of ER tubes from the ONM (figure 6*e*; steps 1 and 2). Fusion of these extensions with the NE (step 3) may entrap cytoplasmic content into ONM encircled compartments (step 4). However, the precise order of these events needs to be established in further experiments.

### 2.7. *APQ12* triggers PA accumulation at the NE dependent on its A*α*H

The experiments above indicate that Apq12 promotes over-proliferation of the ONM and the ER in an A*α*H-dependent manner. A mobilization of lipids by Apq12 would explain the ONM/ER expansion. To test for this possibility, we applied lipid mass spectrometry analysis to evaluate how P_Gal1_-induced overexpression of *APQ12* and *apq12-ah* affected the composition of cellular lipids. Cells carrying the pGal1 construct were used as vector control. Samples were analysed at 0, 1 and 3 h of galactose addition (electronic supplementary material, figure S6A–D). Throughout the experiment, the vector control behaved in a similar manner to *apq12-ah* cells. Interestingly, P_Gal1_-*APQ12-6His* overexpression triggered an increase in DAG and TAG after 1 h of promoter induction (electronic supplementary material, figure S6A). PS and EE in the P_Gal1_-*APQ12-6His* sample was increased after 3 h induction. In addition, the number of two double bonds in GPL decreased in the P_Gal1_-*APQ12-6His* sample after 1 h but with an increase of one double bond after 3 h. Furthermore, P_Gal1_-*APQ12-6His* expression also affected the chain length of GPL after 3 h of induction compared to P_Gal1_-*apq12-ah-6His* induction (electronic supplementary material, figure S6D). Thus, P_Gal1_-*APQ12-6His* expression has a mild impact on the lipid composition of cells.

**Figure 7.**
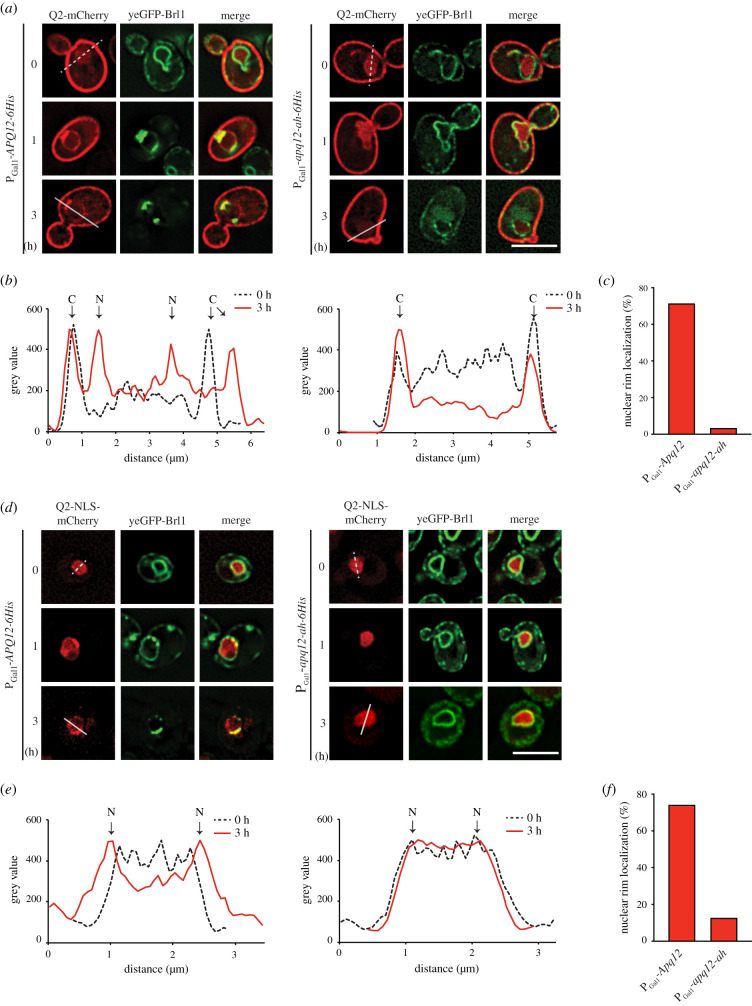
*APQ12* overexpression induces A*α*H dependent PA accumulation at the NE. (*a*) Localization of the cytoplasmic PA sensors (Q2-mCherry) in response to P_Gal1_-*APQ12* and P_Gal1_-*apq12-ah* induction at 0, 1 and 3 h. (*b*) Line scans of mCherry signal along the indicated lines across the cells in (*a*). Arrowheads mark the peaks corresponding to the cytoplasmic membrane (*c*) and NE (N). (*c*) Quantification of data from (*a*) showing percentage of cells with nuclear rim localization after 3 h of Gal induction. 100 cells carrying either P_Gal1_-*APQ12* or P_Gal1_-*apq12-ah* from one representative experiment were analysed for nuclear rim localization of the PA sensor. (*d*) Localization of the nuclear PA sensors (NLS-Q2-mCherry) in response to P_Gal1_-*APQ12* (left) and P_Gal1_-*apq12-ah* (right) induction at 0, 1 and 3 h. (*e*) Line scans of mCherry signal along the indicated lines across the nuclei in (*d*). Arrowheads mark the peaks corresponding to the NE (N). (*f*) Quantification of data from (*d*) showing percentage of cells with nuclear rim localization done as in (*c*). (*a,d*) Scale bars: 5 µm.

Lipid mass spectrometry analysis did not enable us to make conclusions about local changes in lipid content, for example understanding whether the changes were restricted to one location such as the NE or cytoplasmic membrane systems. Using recently reported lipid sensors [8], we tested whether *APQ12* overexpression affects PA accumulation at the INM or ONM, and compared the outcome of overproduction of the wild-type molecule with the impact of *apq12-ah* overexpression. These sensors use the PA recognizing domain of the *S. cerevisiae* transcription factor Opi1, with or without an NLS [8]. We used the cytoplasmic PA sensor Q2-mCherry and nuclear PA sensor NLS-Q2-mCherry for the analysis of PA changes at the ONM and INM, respectively. Consistent with published data [8], at *t* = 0 strong Q2-mCherry decoration of the cytoplasmic membrane (figure 7*a,b*) was accompanied by a weak Q2-mCherry nuclear signal. After 1 h and 3 h of P_Gal1_-*APQ12* induction, the Q2-mCherry signal accumulated in a rim-like pattern at the NE and co-localized with developing yeGFP-Brl1 clusters at the NE (figure 7*a–c*). In P_Gal1_-*apq12-ah* cells at *t* = 0, the Q2-mCherry reporter showed similar localization to P_Gal1_-*APQ12* cells (figure 7*a*) and induction for 1 h did not impact the localization of the Q2-mCherry sensor. After 3 h of induction the nuclear Q2-mCherry signal was nearly completely diminished without directing the sensor to the NE (figure 7*a–c*).

Most interestingly, the nuclear NLS-Q2-mCherry (*t* = 0) was recruited from a nuclear localization at *t* = 0 to a rim-like distribution at the NE 1–3 h after induction of P_Gal1_-*APQ12-6His* (figure 7*d–f*). In addition, NLS-Q2-mCherry strongly co-localized with yeGFP-Brl1 enrichments at the NE (figure 7*d*). By contrast, P_Gal1_-*apq12-ah* expression did not impact the nuclear localization of NLS-Q2-mCherry (figure 7*d–f*). Thus, the A*α*H of *APQ12* is required to induce PA enrichment at the NE.

### 2.8. The A*α*H of Apq12 is not required for the association of the protein with herniations

**Figure 8.**
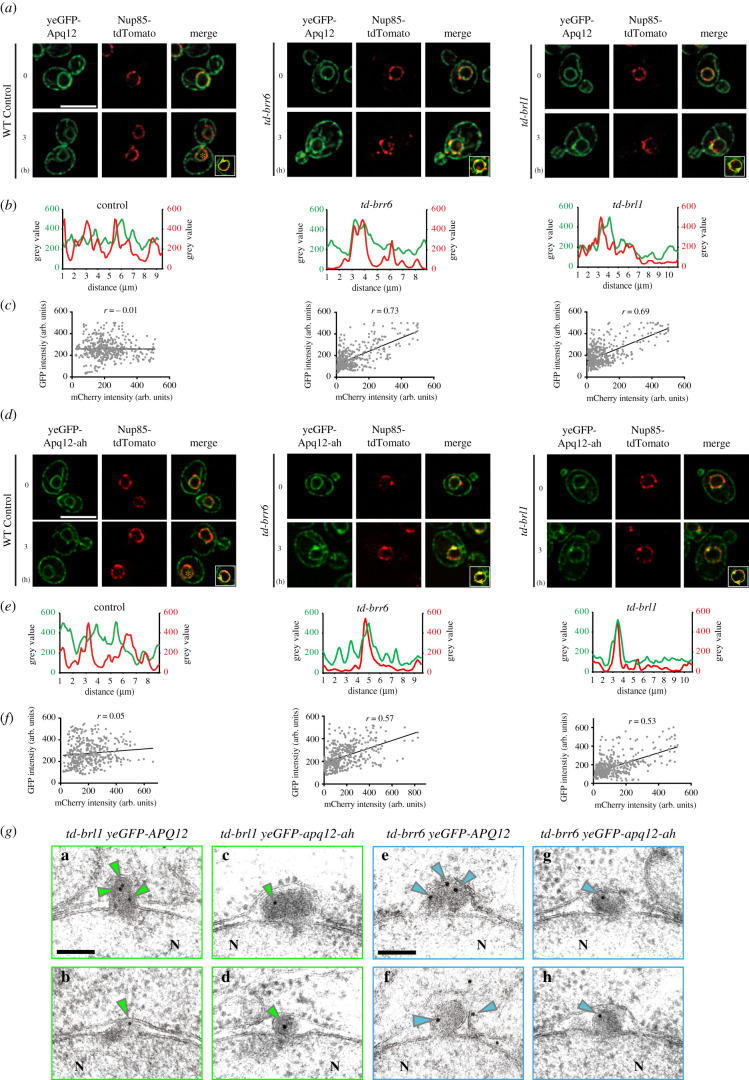
Apq12 associates with NPC biogenesis intermediates. (*a*) yeGFP-Apq12 localization in WT, *td-brr6* and *td-brl1* cells carrying the NPC marker *NUP85-tdTomato*. Cells were incubated for 0 h and 3 h with P_Gal1_-*UBR1* induction at 37°C. (*b*) Line scans along the NE of cells shown in the insets in (*a*). (*c*) Scatterplot with Pearson correlation coefficient (*r*) of GFP and mCherry fluorescence intensities (in arbitrary units, a.u.) along the nuclear rim of 5 cells. (*d*) yeGFP-Apq12-ah localization in WT, *td-brr6* and *td-brl1* cells carrying the NPC marker *NUP85-tdTomato*. Cells were incubated for 0 h and 3 h with P_Gal1_-*UBR1* induction at 37°C. (*e*) Line scans along the NE of cells shown in the insets in (*d*). (*f*) Scatterplot with Pearson correlation coefficient (*r*) of GFP and mCherry fluorescence intensities (in arbitrary units, a.u.) along the nuclear rim of 5 cells. (*a*, *d*) Scale bars: 5 µm. (*g*) *td-brl1* and *td-brr6* cells with *yeGFP-APQ12* or *yeGFP-apq12-ah* were incubated for 3 h under restrictive conditions (galactose, 37°C) to induce degradation of Brl1 and Brr6 and the formation of herniations. Fixed and embedded cells were analysed for localization of yeGFP-Apq12 and yeGFP-Apq12-ah by immuno-EM using GFP antibodies and 10 nm gold labelled protein A. Green and blue arrowheads indicate the localization of 10 nm gold particles. Scale bars: 100 nm.

The co-localization of Apq12 and apq12-ah with a small subset of NPCs (electronic supplementary material, figure S1D) raises the possibility that both proteins resemble Brl1 and Brr6 in associating with the newly assembled NPCs but not fully assembled NPCs [12]. We tested this notion by studying Apq12 and Apq12-ah localization in cells carrying temperature dependent *td-brl1* and *td-brr6* degrons, that accumulate NPC biogenesis intermediates at 37°C [12]. Localization of Apq12 and Apq12-ah at herniations in *nup116Δ* cells was not analysed because of the synthetically lethal phenotype of *nup116 apq12* mutant combination [26]. Interestingly, in *td-brl1* and *td-brr6* cells incubated at 37°C, yeGFP-Apq12 co-localized with Nup85-tdTomato while this was not the case in the equally treated WT control cells, as indicated by the scan of the NE signal (figure 8*a,b*). Quantification of the co-localization of yeGFP-Apq12 and Nup85-tdTomato signals along the NE showed a clear correlation between the Nup85-tdTomato and yeGFP-Apq12 signals in *td-brr6* and *td-brl1* cells, but not in WT control cells (figure 8*c*). We next asked whether a functional A*α*H is required for the co-localization of Apq12 with NPC biogenesis intermediates. In a manner that is reminiscent of yeGFP-Apq12 behaviour (figure 8*a,b*), yeGFP-Apq12-ah co-localized with Nup85-tdTomato in *td-brl1* and *td-brr6,* but not WT cells (figure 8*d,e*). The yeGFP-Apq12-ah and Nup85-tdTomato signals along the NE correlated in *td-brr6* and *td-brl1* cells but not in the WT control cells (figure 8*f*). This together suggests that Apq12 associates with NPC assembly intermediates in *td-brl1* and *td-brr6* cells and that the A*α*H of Apq12 is not required for this localization.

In order to understand the precise localization of yeGFP-Apq12 and yeGFP-Apq12-ah in *td-brl1* and *td-brr6* cells in greater depth, immuno-EM analysis with anti-GFP antibodies was used to determine the subcellular distribution of these proteins. yeGFP-Apq12 was detected at, or inside, herniations (figure 8*g*; electronic supplementary material, figure S7A and B). yeGFP-Apq12-ah was also recruited to herniations (figure 8*g*; electronic supplementary material, figure S7A and B). To ensure that the Apq12 enrichment was not an indirect consequence of the increase in membrane surface in herniations compared to the NE, we measured gold particle number relative to the length of the sectioned membrane along NE and in herniations. This analysis detected less than 1 gold particle per µm of the NE in WT, *td-brl1* and *td-brr6* cells (electronic supplementary material, figure S7C). In herniations, this number was increased to 5 gold particles per µm, clearly indicating an enrichment of Apq12 with herniations.

### 2.9. Interaction of Brl1 and Brr6 is regulated by A*α*H of *APQ12*

**Figure 9.**
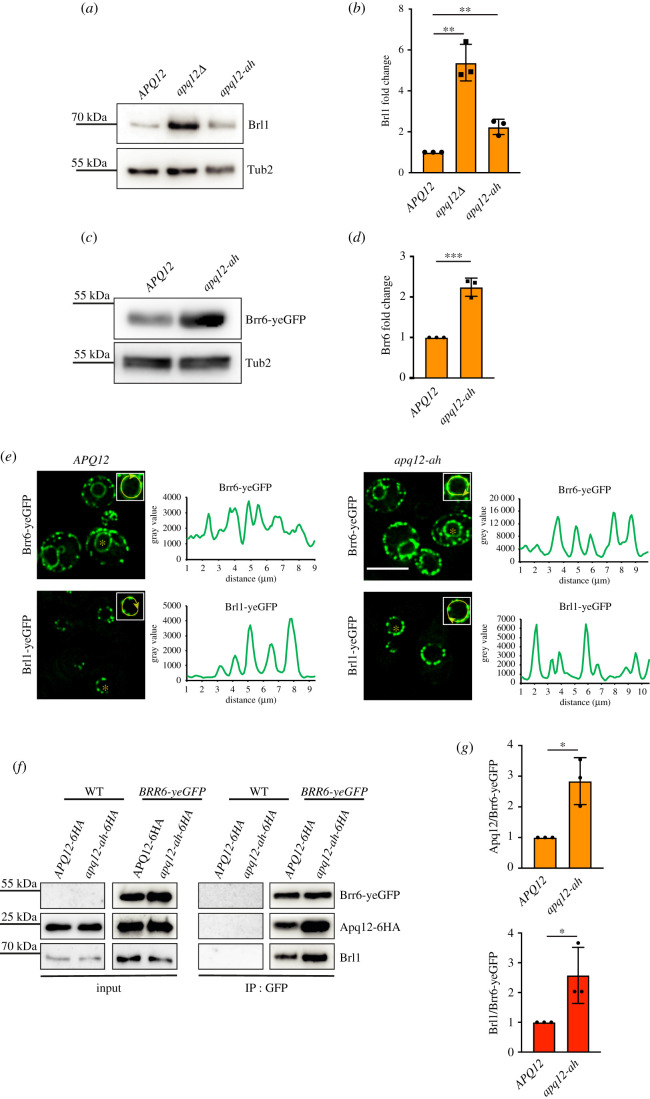
Interaction of Brl1 and Brr6 is regulated by A*α*H of *APQ12.* (*a*) Immunoblot showing endogenous levels of Brl1 in WT *APQ12*, *apq12Δ* and *apq12-ah* backgrounds, using anti-Brl1 antibody. Tub2 is loading control. (*b*) Quantification of Brl1 from (*a*) normalized to Tub2. Error bars are s.d., *n* = 3. **p < 0.01. (*c*) Immunoblot showing levels of Brr6-yeGFP in WT (*APQ12*) and *apq12-ah* backgrounds using anti-GFP antibody. Tub2 is loading control. (*d*) Quantification of Brr6 from (*c*) normalized to Tub2. Error bars are s.d., *n* = 3. ***p < 0.001. (*e*) Localization of Brr6-yeGFP and Brl1-yeGFP in WT *APQ12* (left) and *apq12-ah* cells (right) and corresponding line scans along NE of cells shown in the insets. Scale bar: 5 µm. (*f*) Co-IP of Apq12, Brl1 and Brr6. Brr6-yeGFP was immunoprecipitated with GFP antibodies. Apq12-6HA was detected with anti-HA and Brl1 with Brl1 antibodies. (*g*) Quantification of the ratio between Apq12 and Brr6-yeGFP and, Brl1 and Brr6-yeGFP in WT *APQ12* and *apq12-ah* backgrounds. Error bars are s.d., *n* = 3; *t*-test; *p < 0.05.

Apq12 shows physical and genetic interactions with *BRL1* and *BRR6* [21,23,26]. A dysfunctional A*α*H could have an impact on Brl1 and Brr6 behaviour and their interaction with Apq12. Analysis of the Brl1 protein indicated an increase of Brl1 levels in *apq12Δ* and *apq12-ah* mutants (figure 9*a,b*) compared to WT. This increase was more pronounced in *apq12Δ* mutants (figure 9*a,b*). Because of the lack of Brr6 antibodies, we tested the abundance of Brr6-yeGFP in WT, *apq12Δ* and *apq12-ah* mutants. *apq12Δ* showed lethality in combination with *BRR6-yeGFP* and was therefore not tested. The Brr6-yeGFP level was increased in apq12-ah mutant compared to the WT (figure 9*c,d*). Analysis of the localization of Brl1-yeGFP and Brr6-yeGFP in *apq12-ah* mutants showed cellular distributions of both proteins that were reminiscent of that seen in WT (figure 9*e*), with an increase in signal intensity along the NE and cortical ER in *apq12-ah* mutants (figure 9*e*, grey value of the NE scans). This is consistent with the increase in protein levels of Brl1 and Brr6 in *apq12-ah* cells (figure 9*a–d*).

We next analysed whether the increase in protein levels of Brl1 and Brr6 in mutants is also reflected in Brl1–Brr6 interaction in co-IP experiments in which Brr6-yeGFP was immunoprecipited with anti-GFP antibodies followed by the analysis of the immuno-precipitated proteins with Brl1 and HA antibodies. The Brr6-yeGFP immunoprecipitation showed complex formation of Apq12 and Apq12-ah with Brl1 and Brr6 (figure 9*f*). Quantification of the immunoprecipitated proteins from three independent experiments showed that although Brr6-yeGFP precipitation efficiency was very similar, more Apq12 and Brl1 were co-immunoprecipitated from *apq12-ah* cells than from *APQ12* cells (figure 9*f,g*). Thus, a defect in A*α*H of Apq12 enhances the interaction between Brr6, Brl1 and Apq12.

## 3. Discussion

Apq12 is important for NPC function and cooperates with the essential NPC biogenesis factors Brl1 and Brr6 [21,23,26]. These data indicate that Apq12 is at the heart of machinery that ensures NPC assembly, raising the important question about its molecular function. Because Apq12 does not have any sequence similarities to proteins with enzymatic activity, we have to assume that it functions either as a protein scaffold or impacts the shape of the NE as reported for reticulons that bend the ER by two cooperating pairs of trans-membrane domains with an adjacent A*α*H [37,38]. Following the rationale that a combination of transmembrane domains and A*α*H could be the basis for the function of Apq12, we analysed Apq12 by the AmphipaSeeK program, which predicted the presence of a short AαH connecting the two transmembrane domains. Consistent with this prediction, the synthetic AαH peptide binds to liposomes dependent on its amphipathic nature and the lipid composition. In addition, topological analysis showed that the AαH resides in the intermembrane space of the NE, while N- and C-terminal regions of Apq12 localize in the nucleus or cytoplasm depending on the INM or ONM localization of Apq12.

In terms of cold sensitivity, NE and NPC biogenesis defects, the *apq12-ah* mutant behaves similar to complete loss of *APQ12* function (this study) [21,23,26]. However, phenotypic differences between *apq12-ah* and *apq12Δ* are also detectable (figure 3). In addition, Apq12-ah associates at least as efficiently with Brl1 and Brr6 as Apq12, has the same topological arrangement as Apq12 and associates with NPC assembly intermediates. This together indicates that *apq12-ah* is not a loss of function allele. The A*α*H is required for the overall function of Apq12. However, N- and C-termini of Apq12 fulfil functions without the involvement of A*α*H. This probably explains why prolonged overexpression of *apq12-ah* still shows a mild membrane deforming phenotype (figure 5).

*apq12-ah* and *apq12Δ* mutants impact cellular lipid composition, independent of the tested growth temperature, in a way that reduces membrane fluidity. This accounts for the cold sensitive growth defect and the NE breakdown phenotype at reduced growth temperatures. In addition, we observed the striking accumulation of PA either through synthesis or re-localization at the NE upon overexpression of *APQ12*, dependent on the functionality of the A*α*H. Before, it was shown that PA accumulates at sites of NPC mis-assembly [39] raising the question as to whether PA accumulation at the NE induced by P_Gal1_-*APQ12* overexpression is a consequence of defective NPCs. The observation that P_Gal1_-*APQ12* overexpression merely displaces NPCs into areas that lack ONM expansions without the accumulation of defective NPC assemblies (figure 4*c*; electronic supplementary material, figure S3A), indicates that overproduced Apq12 has the ability to induce PA accumulation at the NE even when NPCs are intact.

Previously we have shown that INM membrane deformations in *td-brl1* and *td-brr6* cells reflect emerging NPCs that do not fully assemble because of a defect in one of the steps leading to the generation of functional NPCs [12]. Using cells carrying *td-brl1* and *td-brr6* we observed strong accumulation of Apq12 with NPC biogenesis intermediates and this localization does not require a functional A*α*H. By contrast, Apq12 only co-localizes with a small number of NPCs in WT cells. Thus, Apq12 joins Brl1 and Brr6 [12] in a small subset of proteins required for NPC assembly that transiently interact with assembling NPCs but then dissociate from NPCs as soon as they are fully assembled.

Like Apq12, Brl1 and Brr6, the Lap2-emerin-MAN1 (LEM) family proteins Heh1 and Heh2 are also nonstructural components of NPCs. However, in contrast to Apq12, Brl1 and Brr6 that function directly in NPC biogenesis, Heh1 and Heh2 contribute to the surveillance of NPC biogenesis [40]. Upon NE damage caused by an NPC biogenesis defect, Chm7, a component of an ESCRT-III like complex, enters the nucleus where it is activated by nuclear Heh1 to promote the sealing of the disrupted NE by the ESCRT machinery [27,40]. In contrast to Heh1, Heh2 probably functions as a senor for the assembly state of NPCs [41]. Thus, Apq12, Brl1, Brr6, Heh1 and Heh2 fulfil quite distinct functions at NPCs.

Based on our findings and the observation that the role of the Apq12-Brl1/Brr6 module overlaps with that of the nucleoporin Nup116 in scaffolding NPC biogenesis [5,12], we suggest the following stages for the stepwise assembly of NPC assembly. The FG nucleoporin Nup116 was shown to scaffold NPC assembly together with Nup188 on the nuclear side of the INM [5,42]. Like Nup116, Apq12, Brl1 and Brr6 also associate with NPC intermediates [12,43]. The interaction of Brl1 with the integral membrane protein Ndc1 [44] and Nup188 could be the trigger for the recruitment of this protein to assembling NPCs [12]. How Apq12 is recruited to NPC intermediates is not understood. The interacting Brl1 and Brr6 are not essential for this localization since Apq12 associates with herniations upon induced depletion of Brl1 and Brr6 (figure 8).

Apq12 probably executes multiple functions, some of which are at least partly mediated by its A*α*H at NPC biogenesis sites. First, Apq12 inserts through its membrane active A*α*H into the NE from within the intermembrane space (figure 1). This insertion has the potential to generate membrane curvature that may stabilize membrane deformations that arise during NPC biogenesis. A*α*H peptide did not deform GUVs under the experimental conditions we applied (figure 1). However, the yeast reticulon Yop1 only deforms liposomes in the context of transmembrane domains and A*α*H [37]. Thus, in future experiments, it will be important to measure the membrane-deforming ability of the TM-A*α*H-TM core of Apq12. Second, the enrichment of Apq12 at NPC biogenesis sites may promote local accumulation of PA as indicated by the *APQ12* overexpression phenotype. Because of its conical shape, PA could stabilize bent membrane regions at INM bends during NPC biogenesis [45]. In addition, PA has the ability to interact with a range of proteins via short stretches of positively charged amino acid residues [46,47]. Presently, no Nup or NPC biogenesis factor with PA binding activity has been described. However, it was only until recently that the PA-binding activity of the ESCRTIII protein Chm7 was reported [39], raising the possibility that proteins involved in NPC assembly may carry hidden PA binding sites. Third, considering that Apq12 has an impact on Brl1 and Brr6 levels and the interaction of Apq12, Brl1 and Brr6 (figure 9), it may coordinate localization of the interacting Brl1 and Brr6 at NPCs biogenesis sites, a function that would be most important at elevated temperatures when Apq12 most strongly plays a role in NPC assembly. *APQ12*, *BRL1* and *NUP116* show genetic interactions and overexpression of *BRL1* is able to suppress the NPC biogenesis defect of *nup116Δ* and *nup116ΔGLFG P_MET3_-NUP188* mutants by promoting the fusion of the INM and ONM [5,12,26]. We therefore suggest that Brl1, in part scaffolded by Apq12, directly or indirectly, facilitates the fusion between the INM with the ONM during NPC biogenesis.

The Apq12-Brl1/Brr6 module is only conserved in organisms with closed mitosis [22]. However, considering the simple domain architecture of Apq12 with two TM domains and a short A*α*H, proteins with similar organization may substitute for the function of Apq12 in higher eukaryotes without having detectable amino acid homology to Apq12, rather a structural similarity with two closely opposed TM domains. Brl1 and Brr6 have an extended intermembrane space domain that is stabilized by two functionally important disulfide bridges [12,22]. This stabilization principal could be substituted for alternative structural features in functional equivalent proteins in higher eukaryotes.

## 4. Material and methods

### 4.1. Yeast strains and plasmids

Plasmids and yeast strains used in this study are listed in electronic supplementary material, table S1. All yeast strains have been derived from ESM356-1 (*MATa ura3-52 trp1Δ63 his3Δ200 leu2Δ1*). A PCR-based integration approach was used for epitope tagging of endogenous genes and gene deletions [48,49]. Yeast strains were cultured in SC (synthetic complete) medium, SC-selection medium [50], YPD (yeast extract, peptone and glucose) or YPRaf (yeast extract, peptone and raffinose) with or without 0.1 mM CuSO_4_ and grown at indicated temperatures. To induce gene expression from the *GAL1* promoter, galactose was added to a final concentration of 2%. TCA precipitations and alkaline lysis were used to prepare yeast extracts for analysing protein levels by western blot. To test for growth defects, yeast cells were grown over night in the indicated medium. 1 OD of cells was collected and then spotted in 10-fold serial dilutions onto selection plates and incubated at indicated temperatures.

### 4.2. EM and immuno-EM analysis of yeast cells

High-pressure freezing, freeze substitution, sectioning, labelling and staining of cells was done as described [51]. Briefly, vacuum filtration and then high pressure freezing with an HPM010 (Abra-Fluid, Switzerland) was used to collect cells onto a onto a 0.45 µm polycarbonate filter (Millipore), followed by freeze-substitution using the EM-AFS2 device (Leica Microsystems, Vienna, Austria) (0.1% glutaraldehyde, 0.2% uranyl acetate, 1% water—in anhydrous acetone) and infiltrated stepwise with Lowicryl HM20 (Polysciences, Warrington, PA), started at −90°C. The samples were gradually warmed up to 20°C for polymerization under UV exposure. Sectioning of embedded cells was done using a Reichert Ultracut S Microtome (Leica Instruments, Vienna, Austria) to a thickness of 80 nm. Post-staining with 3% uranyl acetate and lead citrate was performed. Sections were imaged at a Jeol JE-1400 (Jeol Ltd., Tokyo, Japan) operating at 80 kV equipped with a 4 k × 4 k digital camera (F416, TVIPS, Gauting, Germany). Micrographs were adjusted using ImageJ. For immuno-labelling GFP antibody was used. The samples were prepared in a similar manner but without glutaraldehyde in the freeze substitution solution. Blocking was done in 1.5% BSA, 0.1% fish skin gelatin in PBS, followed by incubation with the primary antibody and treatment with protein A-gold conjugates (10 nm, Utrecht University, Utrecht, The Netherlands).

### 4.3. Fluorescence light microscopy and image analysis

A DeltaVision RT system (Applied Precision, Olympus IX71 based) equipped with the Photometrics CoolSnap HQ camera (Roper Scientific), a 100×/1.4 NA UPlanSAPO objective (Olympus), a mercury arc light source and the softWoRx software (Applied Precision) was used for cell imaging. Imaging was done with exposure times depending on the fluorescence intensity of each protein. For time-lapse experiments, cells were immobilized on Concanavalin A (Sigma-Aldrich)–coated 35-mm glass bottomed dishes (P35G-1.5-14C; MatTek Corporation) and kept in their respective media. Images were deconvolved using the softWoRx software (Applied Precision) and processed with ImageJ (National Institutes of Health, Bethesda MD). For figure 5*c,e*, quantification of maximum intensity of individual cells was done by utilising CellProfiler 3.1.9 software (Broad Institute, Cambridge, MA) [52].

### 4.4. Lipid analysis by mass spectrometry

Cells (10 OD) were homogenized in a FastPrep machine (MP Biomedicals) in 155 mM ammonium bicarbonate buffer pH 7.5 and subjected to acidic Bligh&Dyer lipid extractions in the presence of internal lipid standards containing PC (phosphatidylcholine, 13 : 0/13 : 0, 14 : 0/14 : 0, 20 : 0/20 : 0; 21 : 0/21 : 0, Avanti Polar Lipids), PI (phosphatidylinositol, 17 : 0/20 : 4, Avanti Polar Lipids), PE and PS (phosphatidylethanolamine and phosphatidylserine, 14 : 1/14 : 1, 20 : 1/20 : 1, 22 : 1/22 : 1, semi-synthesized as described [53], DAG (diacylglycerol, 17 : 0/17 : 0, Larodan), TAG (TAG, D5-TAG-Mix, LM-6000/D5-TAG 17 : 0,17 : 1,17 : 1, Avanti Polar Lipids), PA (phosphatidic acid, 17 : 0/20 : 4, Avanti Polar Lipids), PG (phosphatidylglycerol, 14 : 1/14 : 1, 20 : 1/20 : 1, 22 : 1/22 : 1, semi-synthesized [53], and Cer (ceramide, Avanti Polar Lipids). Lipids recovered by organic extraction were evaporated by a gentle stream of nitrogen. Extracted lipids were dissolved in 10 mM ammonium acetate in methanol and transferred to 96-well plates (Eppendorf twintec plate 96). Measurements were performed in positive ion mode on an AB SCIEX QTRAP 6500+ mass spectrometer, equipped with chip-based (HD-D ESI Chip, Advion Biosciences) nano-electrospray infusion and ionization (Triversa Nanomate, Advion Biosciences) as described [53]. The following precursor ion scanning (PREC) and neutral loss scanning (NL) modes were used for the measurement of the lipid classes: +PREC 184 (PC), +PREC282 (t-Cer), +NL141 (PE), +NL185 (PS), +NL277 (PI),+NL189 (PG), +NL115 (PA), +PREC 77 (ergosterol),+PREC379 (ergosteryl ester). Ergosterol was quantified following derivatization to ergosterol acetate in the presence of the internal standard (22E)-Stigmasta-5,7,22-trien-3-beta-ol (Aldrich, R202967) using 100 µl acetic anhydride/chloroform (1 : 12v/v) [54]. Data was analysed using LipidView (ABSciex) and an in-house developed software (ShinyLipids).

### 4.5. Liposome preparation

All lipids were received from Avanti Polar lipids with the exception of Atto647 N, which was obtained from Atto-Tec. The lipid composition of the PM mix consisted of: 34.8 mol% 1-palmitoyl-2-oleoyl-*sn*-glycero-3-phosphocholine (POPC), 15 mol% 1,2-dioleoyl-*sn*-glycero-3-phosphoserine (DOPS), 20 mol% 1-hexadecanoyl-2-octadecenoyl-*sn*-glycero-3-phospho-ethanolamine (POPE), 25 mol% cholesterol (from ovine wool), 5 mol% liver L-α-phosphatidylinositol (PI, from liver) and 0.2 mol% Atto647N-DPPE. The composition of the used NE lipid mix was: 19.8 mol% POPC, 3 mol% DOPS, 42 mol% cholesterol, 7 mol% POPE, 23 mol% PI, 5 mol% PI(4,5)P_2_ and 0.2 mol% Atto647 [55]. SUVs (small uni-lamellar vesicles) were formed as described previous [56] by dissolving the lipid mixtures in Octyl-β-D glucopyranoside (OG) containing buffer, OG dilution below the critical micellar concentration, flow dialysis and SUV isolation using Nycodenz-gradient centrifugation. Subsequently, the concentrated liposomes were extruded 23 times with a 100 nm filter and stored at 4°C.

For preparation of GUVs (giant uni-lamellar vesicles), the PM mix was used. SUVs were prepared to produce GUVs as described previously [57] with the following modifications: (I) the GUVs were desalted two times employing a PD10 column (GE Healthcare) instead of using a Sephadex-G50 gel filtration column in the second desalting step. (II) platinum-coated glass slides (GeSiM) were applied instead of ITO-coated glass slides (GeSiM) [58].

The lipid concentration was measured by the fluorescence of Atto647 N with an excitation of 647 nm and an emission of 670 nm using a Fluoroskan Ascent FL plate reader (Thermo Scientific) in a 96-well plate (integration time: 1000 ms). Therefore, the liposomes were disrupted by 0.5% Dodecyl-maltoside (DDM). The recovery of the total lipid was compared to the lipid after preparation.

### 4.6. Dynamic light scattering of the SUVs

DLS was applied to monitor the size of the liposomes. After adding 2 µM lipid (5 µl final volume) to the quartz cuvette, the particle size was determined in a DynaPor NanoStar (Wyatt Technologies) instrument at room temperature. The buffer composition for size determination was set to PBS and 20 acquisitions with 5 s time intervals were measured.

### 4.7. Binding studies of SUVs

The binding of the A*α*H and A*α*H-ah peptides (from PSL Peptide Specialty Laboratories GmbH, Heidelberg; amino acid sequence: KLLMNFITLVKRFL, KLDMNRNTLVKRNL) coupled with Atto488 (freshly solved in DMSO and diluted in 25 mM HEPES pH 7.4, 135 mM KCl, 1 mM DTT (fusion buffer)) with SUVs was measured by a Nycodenz gradient centrifugation. 0.45 mM lipid was incubated with 18 µM A*α*H or A*α*H-ah peptide or 18 µM Atto488 in 25 mM HEPES pH7.4, 135 mM KCl, 2.1% DMSO and 1 mM DTT for 30 min at room temperature, whereas 10% of the sample was used as the reference input. The peptide without liposomes functioned as a floatation control. Nycodenz (solved in fusion buffer) was added to the sample producing a 40% Nycodenz layer in the bottom of the centrifuged tube. The sample was overlaid with decreasing concentrations of Nycodenz (30%, 20%, 10% and 0%) and centrifuged in an SW55 rotor (Beckman Coulter) 2 h 30 min at 55 000 r.p.m. and 4°C. The floated SUVs were located in the layer between 10%–20% Nycodenz. The recovery of the SUVs was measured by the fluorescence of Atto647 N in the DDM lysed sample and compared to the input. The binding of the peptide or Atto488 was analysed by the fluorescence signal of Atto488 (excitation: 460 nm, emission: 538 nm, integration time: 1000 ms) in this layer and compared to the input probe. The binding of the peptide was corrected to the amount of the floated SUVs.

### 4.8. Binding studies of GUVs

For the peptide interaction to GUVs, 3.3 µM GUVs were incubated with different amounts of peptide in a ratio of 1 : 1 to 10 : 1 in fusion buffer at room temperature for 15 min. As a control analysing the change of the GUV morphology 0.04% DMSO was added to the GUVs without peptide. The morphology of the GUVs was visible in the fluorescence of Atto647 and the interaction of the peptide by the fluorescent signal of Atto488. It was measured in a chambered coverslip (Ibidi, catalogue no. 80826) using a DeltaVision microscope.

### 4.9. MST

For determination of the *K*_D_ values via MST 2 µM Atto488 coupled to A*α*H and A*α*H-ah peptide or Atto488 as a control was incubated 15 min at room temperature with increasing liposome concentrations in fusion buffer with 0.01% Triton ×-100. All samples were filled in Premium capillaries and the thermophoresis was measured in a Monolith NT.115 (NanoTemper) instrument with a LED power of 40% (green filter) and an MST power of 17%. The MST data were evaluated using a single exponential function in consideration of the initial state calculated by linear regression. The data were normalized to the ratio of bound/unbound peptide and the *K_D_* value was determined according to the Hill equation,

$$y=\frac{{\left[c\right]}^{n}}{{\left[c\right]}^{n}+K_{D}^{n}},$$

where *n* = Hill slope.

### 4.10. Immunoprecipitation

25 OD of cells were harvested and resuspended in lysis buffer (20 mM Tris–Cl, pH 8.0, 150 mM NaCl, 5 mM MgCl_2_ and 10% glycerol) containing 10 mM NaF, 60 mM β-glycerophosphate, 1 tablet per 50 ml Roche protease inhibitor cocktail complete (EDTA free), and 1 mM PMSF. Lysis was done in a FastPrep machine (MP Biomedicals) by adding glass beads (BioSpec Products). 0.5% Triton X-100 was added to the cell lysate and incubated on ice for 10 min. The soluble proteins and cell debris were separated by centrifugation and incubated with GFP-Trap agarose beads (Chromotek) for 2 h 4°C. Beads were washed thrice with lysis buffer containing 0.1% Triton X-100 and twice with wash buffer (20 mM Tris–Cl, pH 8.0, 150 mM NaCl and 5 mM MgCl_2_). Elution of bound proteins was done in 50 µl of 2 × SDS-PAGE sample buffer, heated for 5 min at 95°C, and then used for SDS-PAGE and western blotting.

### 4.11. Statistical analysis

For the statistical analyses, PRISM v.7 software (GraphPad) was used. Unpaired *t*-test with two-tailed *p*-value was used to compare samples. Normal data distribution was assumed but formally tested.

### 4.12. Antibodies

Antibodies and their conditions of use are as follows: mouse anti-His (western blot, 1 : 1000; 34660; Qiagen), rabbit anti-Brl1 (western blot, 1 : 1000; made in-house), rabbit anti-Tub2 (western blot, 1 : 1000; made in-house) and rabbit anti-GFP (western blot, 1 : 1000; Proteintech), rabbit anti-HA (western blot, 1 : 500; Proteintech), rabbit anti-GFP (immuno-EM, 1 : 5; gift from M. Seedorf, Zentrum für Molekulare Biologie, Heidelberg, Germany).

## Data accessibility

Additional data are provided in electronic supplementary material [59].
